# How Stress Facilitates Phenotypic Innovation Through Epigenetic Diversity

**DOI:** 10.3389/fpls.2020.606800

**Published:** 2021-01-15

**Authors:** Thanvi Srikant, Hajk-Georg Drost

**Affiliations:** Department of Molecular Biology, Max Planck Institute for Developmental Biology, Tübingen, Germany

**Keywords:** epigenetics (DNA methylation), epigenomics, transposable element, abiotic stress, energy stress, plant engineering, methylome diversity, natural variation in plants

## Abstract

Climate adaptation through phenotypic innovation will become the main challenge for plants during global warming. Plants exhibit a plethora of mechanisms to achieve environmental and developmental plasticity by inducing dynamic alterations of gene regulation and by maximizing natural variation through large population sizes. While successful over long evolutionary time scales, most of these mechanisms lack the short-term adaptive responsiveness that global warming will require. Here, we review our current understanding of the epigenetic regulation of plant genomes, with a focus on stress-response mechanisms and transgenerational inheritance. Field and laboratory-scale experiments on plants exposed to stress have revealed a multitude of temporally controlled, mechanistic strategies integrating both genetic and epigenetic changes on the genome level. We analyze inter- and intra-species population diversity to discuss how methylome differences and transposon activation can be harnessed for short-term adaptive efforts to shape co-evolving traits in response to qualitatively new climate conditions and environmental stress.

## Introduction

Plants grow in a variety of climatic conditions around the world, which we largely attribute to an adaptive genome that evolves over long periods of geological time. However, such extensive diversification requires thousands or up to millions of years, either induced by incremental changes in the local environment or upon sudden exposure to a new terrain (possibly as a result of seed dispersal, human intervention, domestication, etc.). These changes in local or global environment are usually perceived as stress conditions for adapted plant species. For this reason, plants have evolved complex stress response mechanisms and multiple traits as a survival buffer to generate enough plasticity when sudden environmental changes occur. Depending on the environmental cue and the plant species at hand, such mechanisms are either of physiological, epigenetic, or genetic nature and depend on the range, magnitude, and duration of the perceived stress. Whether this perceived stress and its response mechanisms are inherited through multiple generations (stress memory), however, depends on several endogenous and exogeneous factors that we review and examine in detail throughout this article. In particular, we focus on co-evolving traits and co-occurring stress response mechanisms to overcome the limited view of uncoupled stress variables. Additionally, we emphasize the need to introduce gene regulatory networks as a conceptual methodology to study the impact of rapid environmental changes such as global warming on the survival capacity of plant ecotypes or species. This notion of interconnected stress-response mechanisms against multiple external forces acting on plants, and the heritability of these traits can stimulate the development of a new generation of plant engineering tools. We envision that such data can be used to build predictive models able to optimize biotechnological efforts, ultimately for engineering coupled traits and improved stress response mechanisms. To achieve this goal, it is crucial to gain a detailed understanding of the interplay between physiological-, epigenetic-, and genetic- mechanisms in the context of within-individual (**intra**generational) stress responses and the transgenerational manifestation (adaptation) of stress response mechanisms (**inter**generational).

We present a new hypothesis postulating that the physiological level represents a buffer zone that determines the stress response plasticity of a plant within a generation while hedging the epigenetic and genetic levels against stress-induced changes severe enough to be heritable. Only after a stress stimulus overpowers this physiological response buffer, a transgenerationally stabilized stress memory will manifest itself and induce the emergence of new (epi-)genetic stress response variants subjected to natural selection. Furthermore, the epigenetic level itself can also be seen as a buffer zone acting as an interface between the environmental and genetic levels to create a transgenerational buffer while the physiological level represents the buffer zone that determines the adaptive flexibility of a plant species within a generation. We test this hypothesis by reviewing published evidence in support of this notion and provide perspectives on experimental designs for future studies aiming to directly test this conceptual model. A mechanistic understanding of this stress-response interplay is crucial for developing the next generation of plant engineering tools.

In this context, the epigenetic level which can also respond in tandem with stress physiology by selectively reshaping the epigenetic landscape is less likely to manifest these changes transgenerationally, as long as the physiological response is sufficiently strong in buffering the stress stimulus. Once this physiological buffer is saturated, the manifestation of a restructured epigenetic landscape is mostly caused by stress-induced structural variants (SVs) and in particular, transposable element (TE) mobilization that reshuffle genetic material and thereby draw the epigenetic toolkit to new loci while changing the global distribution of epigenetic marks in progeny. However, this genetic reshuffling is only possible when a stress stimulus is strong enough to severely reshape the epigenetic landscape, reactivating previously silenced loci and triggering a mobilization burst to subsequently affect the genetic composition of the germline.

## The Plant Methylome: A Key Player in Stress-Response and Adaptation

Consider a scenario where a generalist plant species is dispersed to a new terrain with increased average temperature levels. In this environment, one form of adaptation could be to slowly increase the basal expression of heat tolerance genes for continuously reducing the impact of this subtle heat ‘stress’ experienced by the plant. Eventually, if the trend of temperature increase continues over several generations, these regulatory changes will be ingrained and fixed on the genetic level.

Yet, a plant that has to survive in a suddenly changing environment such as global warming where both the temperature and precipitation are drastically different and the salinity concentration of the soil increases rapidly, would need to alter several genes and pathways in tandem to maintain reproductive fitness. For most species, this would certainly be a stronger ‘accumulative stress’ experienced by the individual plant and requires more complex gene regulatory re-wiring for short-term adaptation. When experiencing accumulative stress, one possibility for the plant is to employ a combination of epigenetic and genetic mechanisms to maintain genome integrity without the risk of being subjected to several gene regulatory trade-offs when major physiological or developmental changes occur simultaneously. In this article, we examine the various outcomes of the interplay between epigenetic, genetic and physiological regulatory networks by first reviewing our current understanding of stress response mechanisms in plants and then providing future perspectives and applications within the (stress) epigenetics and plant engineering fields. In particular, we focus on DNA methylation as an epigenetic mark involved in gene expression regulation, and how these marks can trigger a cascade of effects that favor both short term (epigenetic response) and long term (transgenerational manifestation) adaptation to stress.

DNA methylation marks (at 5’ cytosine positions) are epigenetic signatures encoding the state of a genome exposed to particular endogenous (e.g., developmental) and exogenous (e.g., environmental) factors throughout intra- and inter-generational time. A subset of these marks are sometimes closely dependent on histone modifications, and are often accompanied by other epigenomic features such as histone variants and chromatin accessibility (Lippman et al., 2004; Johnson et al., 2007; Slotkin and Martienssen, 2007; Li et al., 2018). Overall, DNA methylation marks have genome-wide distribution patterns that can be reshaped upon exposure to environmental stress.

With regards to DNA methylation, such genome-wide changes are generally observed as differentially methylated regions (DMRs) that occur in tandem with transcriptomic or other epigenetic changes under a certain condition. These marks can also be found as heritable natural epigenetic alleles (epialleles) (Schmitz et al., 2013). The methylation signatures of many such epialleles have the potential to fine-tune the expression of flanking genes or certain transposable elements based on their methylation levels. Natural and spontaneously occurring epialleles have been identified in several plant species (Weigel and Colot, 2012). Tracing the evolutionary origins and transgenerational manifestation of these marks has been expedited by the availability of large methylome datasets, some of which have recently provided evidence for the emergence of epialleles from diverse mechanisms of maintaining methylation homeostasis in natural strains (Zhang et al., 2020).

Since population diversity is largely governed by changes in the environment, a large spectrum of environmental stresses have been studied under controlled laboratory conditions. These include biotic and abiotic stresses which are strongly experienced both above and below ground level (salinity, nitrogen and phosphate levels, pathogens, temperature, light exposure and drought, etc.).

While only a subset of all methylome signatures may directly trigger short-term stress response mechanisms, there is evidence that methylation changes are accompanied by other epigenetic changes that in turn can drive an adaptive process benefiting the plant in the long run (Fang et al., 2017; Reynoso et al., 2019; Forestan et al., 2020). Technological advances in sequencing approaches have facilitated the discovery of candidate gene-regulatory elements throughout the genome that may be conserved across strains and species. These elements are often hotspots for a combination of epigenetic marks that include DNA/histone methylation, unique histone variants, accessible chromatin regions and topologically associated domains (TADs) (Maher et al., 2017; Lu et al., 2019; Ricci et al., 2019; Karaaslan et al., 2020).

Together, the tight interplay between plant stress physiology, epigenomic regulation, and adaptive evolution requires a new focus in the light of rapid shifts in global environmental conditions such as climate change. Learning from the examples presented in this work, we propose future directions for plant stress tolerance engineering that harness the naturally occuring activation potential of transposable elements (TEs) (Paszkowski, 2015; Benoit et al., 2019) and natural variants of the methylation apparatus derived from ecotype or species comparisons (Schmid et al., 2018) to facilitate adaptive innovation in response to qualitatively new climate conditions.

## Can Stress-Response Physiology Hedge Transgenerational Methylome (In)Stability?

When sudden or severe changes occur, plants which usually grow in a constant environmental niche can overcome their basal physiological response mechanisms and induce transgenerationally stable changes to the epigenetic landscape (Baulcombe and Dean, 2014). Such changes (genetic and epigenetic) can alter gene expression in order to improve the adaptive fitness of the plant upon the threat of death and ultimately population extinction. Additionally, the epigenetic landscape can be even more dynamic when genetic material is reshuffled (SVs and TEs) as a consequence of the applied stress. It is well known that structural variants in several plant species can generate wide phenotypic diversity (for example, The 1001 Genomes Consortium, 2016; Zhou et al., 2019; Alonge et al., 2020) and some of which can enable improved stress tolerance, recently demonstrated by studies such as Kalladan et al. (2017), Catacchio et al. (2019), Picart-Picolo et al. (2020). Yet, genetic mutations in methyltransferase enzymes for tuning epigenetic stability (Shen et al., 2014; Sasaki et al., 2019), or heritable genome rearrangements catalyzed by TE insertions can contain the necessary stress-response DMRs that may function to either deteriorate fitness or for adding a newly acquired stress tolerance that allows future generations to adapt to the altered environmental condition (Quadrana and Colot, 2016).

Although comprehensive evidence of the inheritance of environmentally induced changes in DNA methylation remains to be collected (Quadrana and Colot, 2016), emerging evidence suggests that epigenetic variation can be exposed to natural selection and induce rapid adaptive responses (Schmid et al., 2018). It remains to be explored whether environmentally induced epigenetic variation is a rare event violating the principles of epigenetic homeostasis (Williams and Gehring, 2020) or whether such events occur frequently enough in large populations to provide a mechanism for rapid adaptation as theoretical population genetic models suggest (Pal, 1998; Pál and Miklós, 1999; Day and Bonduriansky, 2011; Geoghegan and Spencer, 2013a, b; Jablonka and Lamb, 2015; Kronholm and Collins, 2016) and reviewed in Quadrana and Colot (2016). Here we assume that future experiments will unveil more examples of environmentally induced heritable changes to epigenomic landscapes. These insights will raise further questions regarding the association between physiological responses and epigenetic remodeling whereby physiology could act as a buffering layer before environmentally induced heritable changes to epigenomic landscapes can manifest themselves transgenerationally.

To differentiate various stress-response mechanisms and their impact on epigenetic landscapes in plants, we classify stresses by their ability to restrict energy and nutrient supply to the plant body. In this context, abiotic stresses fall under the class of energy-depleting and starvation stresses (Baena-González et al., 2007; Baena-González and Sheen, 2008; Mason et al., 2014), whereas biotic stresses can in particular cases be associated with a more complex energy housekeeping balance (for example parasitic relationships) (Alvarez et al., 2010). We believe that this distinction is vital for plant stress perception, and ultimately determines which cohort of response mechanisms will be employed when coping with respective conditions. This energy notion of stress response predicts a hierarchy of stress tolerance whereby the availability of energy determines the extent to which plants can explore their full spectrum of response mechanisms. This perspective would predict that starved plants will focus energy supply to the most vital response pathways and thereby be exposed to more dramatic epigenetic modifications in comparison with fully nutriated plants that could employ a broader spectrum of physiological responses to buffer the impact on their epigenomic landscape. Although this link between convergent energy-stress and epigenetic remodeling remains to be further explored in plants (Hauben et al., 2009; De Block and Van Lijsebettens, 2011; Ljung et al., 2015), evidence in support of this idea has been accumulated in *Drosophila* (Riahi et al., 2019). From an ecological perspective it is well established that the global availability of food/energy supply determines the reproductive fitness and thereby population size of a species (Darwin and Wallace, 1858). It remains to be established, however, to what degree and at what speed individuals within a population can evolve (epi-)genetic stress-response variants to survive under rapidly changing environments, either under high inputs of food/energy supply or during periods of starvation. Finally, we would like to point out that extensive mechanistic studies connecting energy balance, physiology, and epigenetic remodeling are largely lacking and are only emerging for some model organisms. However, we do see an exciting opportunity in the coming years to employ multi-omics approaches to further investigate these complex relationships.

## Sensing Stress at the Grassroots: How Physiological Responses Prevent or Enable Heritable Methylation Marks

Soil is a major source of nutrition for the sessile plant - it needs the right properties of pH, osmolarity, water, micro- and macro-nutrients to favor reproductive fitness and adaptability. Salinity levels for example, exhibit high variability across plant habitats ranging from deserts to marshlands and are often studied in the context of physiological and gene-level stress response (Bui, 2013). Since salinity is a property that also changes gradually alongside new climate conditions, it can be perceived as a plant stress that requires epigenetic acclimatization.

A transgenerational study in *Arabidopsis thaliana* (Wibowo et al., 2016) showed that seedlings grown in highly saline growth media carry stress-induced DMRs, a subset of which are passed on to their progeny through the female germline. Yet, these DMRs maintain their methylation state only as long as the saline conditions remain constantly present, reverting to their wild-type state when this stress no longer exists. These results raise the question: what factors govern the plasticity of the underlying stress-response methylomes, and the sensitivity with which the saline stress is perceived on the epigenetic level? While the exact threshold for this saline sensitivity (weighed by time or the strength of the applied stress) remains undetermined, there is evidence to show that epigenetic changes do get fixed over time, establishing signatures specific to the climatic condition experienced by the entire population. For example, differences in soil salinity arising from tidal water and nutrient circulation have also been shown to contribute to epigenetic diversity between salt marsh and riverside mangrove populations in Brazil (Lira-Medeiros et al., 2010). Interestingly, these populations are differentiated by genetic mutations as well (fewer in number than the epimutations), but it remains unknown if these mutations also affect the methylation machinery at the intra-species level.

Natural varieties of olive (*Olea europaea*) have been shown to be differentially tolerant to salinity (in a hydroponic system), as observed by phenotypic and metabolic changes (Mousavi et al., 2019). Although both salt-susceptible and salt-tolerant cultivars exhibit an overall increase in methylation levels upon salt stress, the susceptible cultivars harbored hypomethylated loci flanking upregulated genes involved in ionic exclusion, water and nutrient uptake. These results are in contrast with studies in rice, where salt tolerant varieties exhibit genome-wide demethylation as opposed to susceptible varieties (Ferreira et al., 2015). Methylation changes in the tolerant lines could be linked to stress-induced expression changes in demethylase and methyltransferase genes. It appears that different species (and subspecies) have evolved distinct mechanisms (such as altering the expression of stress-response genes through the methylome, or the methylation machinery as a whole) for enabling metabolic responses to this stress. A transgenerational experimental design to address the aforementioned studies would provide more insight on how these distinct mechanisms affect the germline and are fixed over evolutionary time.

Apart from natural sodium and potassium salts in water, depletion of soil nutrients can also pose as a type of stress detrimental for plant growth. Secco et al. explored the epigenetic and transcriptomic responses of plants starved of inorganic phosphate in soil (source for the vital macronutrient Phosphorus), in an attempt to resolve and compare the temporal hierarchy of stress response mechanisms between rice (*O. sativa*) and *A. thaliana* plants (Secco et al., 2015).

The study found that DMRs under phosphate starvation were more predominant in the rice genome compared to *A. thaliana*. This observation fits with genomic structural differences between the two species, especially with regards to transposable elements (TEs). TEs are mobile genetic elements that can change their genomic location by either exploiting a cut-and-paste mechanism (retro-elements) or by increasing their copy number via a copy-and-paste mechanism (DNA elements) (Feschotte, 2008). Most of the DMRs under phosphate starvation were hypermethylated in the CHH context, silencing TEs upon long-term stress and found to be flanking highly induced phosphate-homeostasis genes (Secco et al., 2015). From additional results that indicate the precedence of gene expression over methylation changes, the authors propose that TE activation may be a by-product of proximal gene transcription, eventually being silenced by *de novo* methylation. Yet, these DMRs are not transmitted meiotically, which leads us to ask whether high TE loads are an adaptive advantage or disadvantage for evolving genomes. The rice genome, for example, could harbor several TEs to provide a buffer against stress and avoid some potentially lethal methylation changes in the genome, yet would also be equally prone to the deleterious effects of chromatin reorganization during the brief activation of particular TE families. A TE-poor genome such as *A. thaliana* would presumably have other genetic/epigenetic or even physiological mechanisms to respond to this stress, which explains the reduced number of DMRs.

While we can monitor the mechanistic changes that occur over temporal scales, the intricate steps involved in instantaneous stress perception and the hierarchical order of cellular events leading to the manifestation of epigenetic marks, are still poorly explored. If we already know how DNA and RNA-level modifications occur upon stress, how do they interact with sensory and hormonal signaling networks?

In this context, some studies show that biotic stress can induce DNA demethylation and activate the expression of certain TEs and defense genes (including NLRs), possibly by interacting with pathogen-responsive elements located in gene promoters (Yu et al., 2013). Revisiting the example of salt stress in soils, there is further evidence that altered salt concentrations can be sensed by plant roots, which modulate their growth rates by accumulating reduced auxin levels. In rice, adaptation to saline soils has been attributed to altered GA (Gibberellic Acid) levels during different stages of growth (as reviewed by Yu et al., 2020). Further studies have shown that the expression of histone acetylases and deacetylases can be influenced by hormonal cues such as ethylene and jasmonic acid signaling, and indirectly regulate auxin response pathways (Song et al., 2005; Zhou et al., 2005). Similarly, histone methylation and DNA methylation are linked with the expression of auxin efflux proteins such as PIN1, one classic example being the crosstalk between non-coding RNA, chromatin looping and H3K27me3 at the *APOLO* locus in *A. thaliana*, which is dynamically regulated upon changes in auxin concentration (Ariel et al., 2014, 2020). Chromatin remodeling proteins such as PICKLE and BRAHMA in *A. thaliana* which employ their ATPase activity to alter nucleosomal structure, can also affect accessibility to transcription factors and thereby gene expression of carrier proteins involved in ABA, GA, CK and auxin (IAA) synthesis pathways. *pkl* mutants are not only hypersensitive to salt, chilling and freezing stress (Yang et al., 2019), but also hypersensitive to germination upon abscisic acid (ABA) treatment (Perruc et al., 2007), demonstrating their strong interdependence with hormones regulating plant growth and development.

Although there is still no clear characterization of a temporal hierarchy in stress response and their impact on the epigenetic landscape, these findings certainly illustrate the possibility of hormonal signaling networks initiating genomic and epigenomic changes, but also being tightly feedback-controlled by chromatin.

## Biotic Stress Response: Switching Between Epigenetic Defense and TE Activation

While natural strains in *A. thaliana* show large differences in their methylomes (Kawakatsu et al., 2016) epiRILs (epigenetic Recombinant Inbred Lines) generated from crosses between hypomethylated mutant lines (such as *met1, ddm1*) and a wild-type strain, carry a mosaic of DMRs originating from their parents. epiRILs provide a useful germplasm collection to mine for methylation variants or epialleles that influence gene expression for a desirable trait. The uniquely recombined methylation signatures harbored in individual epiRIL lines result in large phenotypic variation, possibly due to the mosaicity of epigenomes and mobilized transposable elements. This can be observed, for example, in progeny of *met1-3* mutants and wild-type plants propagated over several generations. When infected with *Pseudomonas syringae pv.* tomato(*Pst*), a subset of these lines show increased resistance or susceptibility compared to their parental lines, representing approximately 58% of the resistance variation in 127 natural accessions of *A. thaliana* (Reinders et al., 2009). It will be interesting to examine whether the subset of DMRs, or the epialleles determining the resistance phenotypes are shared between the epiRILs and natural accessions, providing clues to understand the degree of inbreeding methylation stochasticity that governs methylome heritability. Curiously, the methylation state of these epiRILs also activates the transposition of the CACTA transposon family, which is silenced in *met1-3* mutants, indicating that epigenomic recombination can trigger interaction effects that may affect select loci *in trans*.

Furci et al. (2019) used Col-0 x *ddm1* epiRIL lines in *A. thaliana* to identify genomic regions that confer methylation-mediated resistance to biotic stress by the downy mildew pathogen *Hpa* (*Hyaloperonospora arabidopsidis*). Although several epiQTL loci were identified from the varying quantitative resistance between lines, regions that were epigenetically primed by the stress and inherited in the F10 generation were also found outside these associated loci. It was found that these primed DMRs largely overlap with TEs or TE- related genes and likely regulate *in trans* the expression of *Hpa*-resistance and defense genes. When a similar *Hpa* infection is introduced in *A. thaliana* mutants of various proteins involved in DNA methylation, systemic acquired resistance to the infection is impeded from transgenerational carry-over (Luna and Ton, 2012). Most notably, it was found that hypomethylation in the CHG context catalyzed by the KYP and CMT3 proteins could be crucial for generating transgenerational memory in this pathogen species.

Biotic stresses can also induce TE-specific methylation changes, such as during *Pseudomonas syringae* pv. tomato (*Pst*) infection in *A. thaliana.* Cambiagno et al. examined whether epigenetic induction of *PRR/NLR* genes affects pericentromeric TE expression during infection (Cambiagno et al., 2018). Indeed, four TE-families (belonging to the LTR-Gypsy Superfamily) are activated upon infection triggered by hypomethylation at these loci, along with similarly induced *NLR* genes. Surprisingly, prolonged infection recruits siRNAs directed against both sets of loci, thereby eventually re-silencing them with RdDM methylation. Furthermore, the authors showed that a *mom1* (*Morpheus’ Molecule-1*) mutant in which some pericentromeric TEs are expressed also activates the expression of unlinked *PRR/NLR* genes, priming these plants against *Pst.* infection. The cohort of sRNAs that commonly regulate both distal TEs and non-specific *NLR* genes upon re-establishment of methylation marks hint at the potential for transposable elements as triggers for initiating genome-wide immune responses, although the exact mechanisms remain unclear. From the plant’s perspective, controlling multiple loci carrying similar genetic/epigenetic motifs may be efficient, but this could also be facilitated by tuning a single master regulator causing diverse downstream outcomes.

## Control vs. Chaos: Reorganization of the Genome and the Epigenome Under High Temperatures

When the need arises for rapid genome transformation in response to stress, TEs can function as master regulatory switches, catalyzing a domino effect on the somatic and meiotic epigenome upon their mobilization and reintegration. One of the most seminal discoveries in the epigenetics of plant heat response is that of the *ONSEN* (*ATCOPIA78*) family of TEs in *A. thaliana* (Ito et al., 2011). When mutants defective in siRNA biogenesis, such as *nrpd1*, are subjected to heat stress at 37°C, the *ONSEN* family of LTR elements are transcribed, extra-chromosomally replicated and re-inserted into various loci (a detailed mobilization protocol can be found at Gaubert et al., 2017; Sanchez et al., 2017). Furthermore, repeating such a heat treatment in two consecutive generations results in additional reactivation of the newly inserted elements, only to create more copies genome-wide (Matsunaga et al., 2012). It was recently shown that heat stress can also induce dispersion of the constitutive heterochromatin in canonical RdDM mutants (*nrpd1*, *rdr2, drm2*) which could potentially contribute to higher transposition rates and increased copy number of *ONSEN* elements (Hayashi et al., 2020).

Delving further into possible links between chromatin and DNA methylation, Quadrana et al. (2019) examined the locations of new TE insertions in *nrpd1* mutants under 37°C heat stress, to find that the insertion sites were largely found in proximity to the histone variant H2A.Z (Quadrana et al., 2019). The authors showed that epiRIL lines derived from a *ddm1* x Col-0 cross also display a similar enrichment for H2A.Z near insertion sites of *ATCOPIA93*. This belongs to one of three TE families that harbored the largest number of private insertions examined in 107 F8 epiRIL lines. Similarly, the *VANDAL21* family of insertions were enriched for DNaseI hypersensitivity sites (having accessible chromatin) and the *ATENSPM3-* family insertions enriched for H3K27me3 (histone methylation). Taken together, these results are indicative of the specificity with which certain TE families re-integrate their copies into the genome, preferentially favoring chromatin marks that lie within genes involved in environmental stress response.

What would be the consequences of TE mobilization in epiRILs derived from naturally hypomethylated strains? Take for example, Cvi-0, a strain that is largely hypomethylated among the 1001 sequenced strains of *A. thaliana* (Kawakatsu et al., 2016). It is tempting to speculate that genome-wide hypermethylation or hypomethylation would exhibit extreme ranges of epigenetic flexibility for facilitating stress-response. Alternatively, more robust stress resistance mechanisms at the epigenetic level may be the result of a combinatorial effect of chromatin marks and a methylation landscape determining the resistance effect rather than a uniformly high methylation level. It remains to be explored which of these scenarios is the guiding principle of epigenetic stress tolerance, but some studies started to address these questions from various angles (Tittel-Elmer et al., 2010; Mirouze and Paszkowski, 2011; Mirouze et al., 2012; Iwasaki and Paszkowski, 2014a, b; Ito et al., 2015; Hosaka and Kakutani, 2018). From the *ONSEN* example, it appears that multigenerational stress in such a hypomethylated background would only be more deleterious for the genome although recent evidence suggests that transgenic lines of hypomethylated poplars show higher tolerance to water deficit (Sow et al., 2020) hinting toward more complex mechanisms involved in this process. Furthermore, it remains unclear whether the locations of new copies (re-insertions) will remain specific to H2A.Z marks and genic loci (Gaubert et al., 2017) over consecutive generations, or eventually generate a more stochastic genome-wide pattern.

In epiRIL lines of *met1*, the activation of the mobile retrotransposon copy *EVADE* reaches a saturation point of approximately 40 copies in the F10 generation, after which siRNA-induced silencing is restarted once again (Marí-Ordóñez et al., 2013). This suggests a possible mechanism for the cell machinery to recognize TE load and prevent further gene disruption. Unlike *ONSEN*, *EVADE* (Mirouze et al., 2009) can be activated by hypomethylation alone - which means that progeny carrying the hypomethylated epialleles can continue carrying this activated TE in the next generations. *ONSEN* elements, on the other hand, can get transcriptionally silenced by methylation upon re-integration and would only reactivate in the progeny upon continuous heat stress application.

Apart from examples of heat-activated TEs in *A. thaliana*, a newly discovered giant retro-element named *MESSI* in the tomato genome has recently been identified upon long-term heat exposure. This element can potentially exploit developmentally associated escape strategies during tomato meristem development and overcome transcriptional gene silencing in vegetative tissues, thus inducing genetic variation in progeny plants (Sanchez et al., 2019).

The methylome landscape under high temperatures may also be controlled at the genetic level, driven by structural variations at methyltransferase genes, such as *CMT2* in *A. thaliana* (Shen et al., 2014). *cmt2* mutants and accessions carrying a natural knockout (*CMT2-STOP* allele) also exhibit an increased tolerance to high temperatures, resulting from CHH hypomethylation that alters gene expression for stress-response. Ultimately, the tight inter-dependence between genetic and epigenetic pathways makes it challenging to identify which of these factors were first established during the evolutionary adaptation to temperature changes.

## To What Extent Does Magnitude of Stress Determine the Magnitude of Response?

It is important to note that most of the above studies examine the effects of sudden and drastic stresses on plants, which may not always reflect natural settings. In wild plants, it remains to be explored how epigenetic plasticity comes to the rescue when the plant experiences a gradual change in its environment over time. Taking the example of drought stress, it was recently shown that mild conditions of water deficit in *A. thaliana* only trigger minor changes to overall methylation patterns (Van Dooren et al., 2018). Moreover, only 2 out of 468 genes with altered expression under such a drought stress, were associated with differentially methylated positions. While there is some degree of strain-level differentiation in response to the stress (measured by reduction in rosette area), this epigenetic and transcriptomic plasticity is not inherited in the progeny. An earlier study that examined mild drought stress over 5–6 generations in *A. thaliana* came to the same conclusion regarding the transgenerational stability of the methylome, and the rare occurrence of heritable phenotypic changes (Ganguly et al., 2017). In maize however, mild drought triggers transcription of long non-coding RNAs and histone methylation changes that can be retained in the germline as a type of stress-memory (Zhang et al., 2014; Forestan et al., 2020).

While it has been shown that polygenic architecture and loss-of-function alleles can explain differential tolerance of *A. thaliana* accessions to drought (Exposito-Alonso et al., 2018; Monroe et al., 2018), heavy drought stress response remains to be examined at the methylome level. Different strains of *citrus* plants on the other hand, have shown varied responses both in physiology as well as methylation levels to repeated cycles of water-deficit (Neves et al., 2017). Repeated drought exposure in 11 consecutive generations of two rice varieties significantly improved their adaptability, accumulating epimutations specific to stress-response genes (Zheng et al., 2017), while in tomato drought-stress response has been linked to the activity of the retrotransposon family *Rider* (Benoit et al., 2019). Since the nature of the stress may hold varied importance across species for the plant’s developmental physiology, together this could possibly explain the differences in methylation changes attributed to genotypic identity and the deviation in water supply beyond optimal levels.

## Examining the Consequences of Stress During the Epigenetic Reset in Early Embryogenesis

In angiosperm plants, DNA methylation, histone methylation and other chromatin marks play key roles before and after double-fertilization, thereby ‘resetting’ the epigenome of the developing embryo. It is known that DNA methylation in the male gametes catalyze the production of TE-derived siRNAs, accompanied by chromatin changes in the female megaspore mother cell (MMC), eventually forming the endosperm and the embryo. The endosperm is largely hypomethylated compared to the embryo, resulting in the expression of several maternal epialleles (imprinted genes) and also paternally expressed genes (activated by histone methylation in their maternal alleles) (as reviewed by Wang and Köhler, 2017). These are only a few examples of the complex epigenetic dynamics that determine the fitness of the progeny.

Given the complexity of methylome response to stress, can the altered epigenetic state of individual loci during fertilization be sufficient to determine fitness levels across strains? Taking imprinting as an example, the specificity of demethylases such as DME and ROS1, in tandem with DNA and histone methyltransferases could catalyze a domino effect in impeding healthy development of the embryo and the endosperm. In *A. thaliana* for example, differential methylation of the imprinted epiallele *HDG3* in natural accessions can negatively impact embryo development and seed size (Pignatta et al., 2018), demonstrating the power of such unique loci in determining adaptive fitness.

In the context of stress, the germline plays a crucial role in maintaining the integrity of the genome and passing on the DMRs that are necessary for defense. A subset of these DMRs could also overlap with TEs, activating them in the process. In epiRILs of *met1* for example, *EVADE* transcription was observed in the L2 subepidermal adaxial layer of cotyledons, indicating transmission of this element through the female placental organs (Marí-Ordóñez et al., 2013). It is possible that post-meiotic methylation changes can activate silenced *EVADE* copies genome-wide, thereby generating genetic diversity even within seeds of the same silique.

What are the consequences of by-passing the epigenetic reset that occurs during fertilization? Wibowo et al. addressed this question by examining somatic regenerant lines from various postembryonic organs in *A. thaliana* (Wibowo et al., 2018). Their findings reveal that root-tissue derived regenerants heritably retain many root-specific methylome and transcriptome signatures not only in their roots but also their leaves. This indicates the importance of meiotic and post-fertilization processes in determining tissue identity in the growing plant.

When generating hybrids, fertilization processes involve a foreign species or strain where a cohort of homologous epigenetic marks and enzymes also interact with each other. Although this process may be successful in generating healthy seeds, successive generations may bear a negative impact of such hybridization stress. Tomato hybrids, for example, show the gradual transgressive accumulation of siRNAs indicative of epistatic epigenetic interactions between parental marks, thus generating phenotypic diversity (Shivaprasad et al., 2012). There are examples where these epigenetic clashes may be briefly beneficial, such as intra-specific hybrids in *A. thaliana* that show more immediate and predominant epigenetic effects contributing to hybrid vigor (Groszmann et al., 2011). The authors of this study also propose that reduction of hybrid vigor upon segregation may be attributed to the ‘balancing’ of parental epialleles and eventually reducing epigenetic diversity. Epialleles governing hybrid incompatibility, such as the histidine biosynthesis gene *HISN6B*, the folate transporter *FOLT1* and *TAD3* have also been discovered in natural *A. thaliana* accessions (Durand et al., 2012; Agorio et al., 2017; Blevins et al., 2017).

## Future Perspectives: Harnessing Epigenetic Diversity at the Population Level

In recent years, research on intra-specific variation in plants is gaining more prominence while benefiting from previous work on inter-specific variation. Intra-specific variation encompasses strain-level differences of the same species acclimatized to diverse habitats, while variation between species can be useful to study a broader picture of independently occurring evolutionary trajectories that are largely divergent from each other. Research is now focused on understanding and quantifying the degree of changes at the genetic and epigenetic level within populations (Kawakatsu et al., 2016; Quadrana et al., 2016; Stuart et al., 2016; Lanciano and Mirouze, 2018).

While large-scale sequencing of 1001 phenotypically distinct strains in *A. thaliana* has revealed significant differences in population structure (Kawakatsu et al., 2016), it has also brought to light how gene expression, regulatory marks, and repetitive elements can optimally organize their interactions to enable fitness under diverse climatic conditions around the world. This has paved the way for research on strain-level differences across other plant species, such as agriculturally important crops, especially in the light of climate change dependent domestication. Some of these new questions are: To what extent can epigenetic variants drive domestication or speciation? How far do the genome and epigenome mold themselves to maximize their adaptive advantage while maintaining species-specific identity?

**FIGURE 1:**
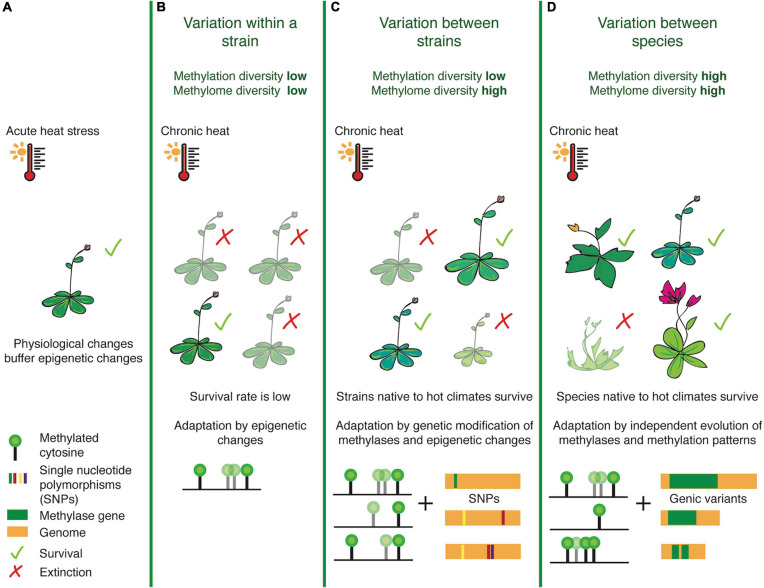
Illustrating the differences between methylation and methylome diversity at the population, strain and species-levels. Taking the example of heat stress, physiological changes often occur as an immediate response to acute changes in temperature before epigenetic pathways and transgenerationally stable changes in epigenetic landscapes fall into place **(A)**. When the stress is chronic, populations belonging to a strain **(B)** non-native to the harsh temperature change often exhibit low survival rates, as they have rather homogenous methylation patterns and can rely only on rapid epigenetic/epigenomic changes for rapid stress-response mechanisms. Between strains however **(C)**, methylome diversity is high and this may enable survival for plants harboring heat-resistant epialleles and modified methyltransferases. At the species-level **(D)**, plants exhibit high levels of methylation and methylome diversity, due to evolutionary fixation of genetic and epigenetic marks, thereby providing an edge for heat-primed species to adapt with greater ease.

Focusing on diversity and natural variation at the level of DNA methylation, we attempt to re-define ‘methylome diversity’ as a metric that measures cytosine methylation in the 3-nucleotide context (CG, CHG, CHH), linked stoichiometrically with the percentage of methylation levels. While this definition does not capture the exact origin of diversification (e.g., TEs or SVs), it will allow us to determine DMR hotspots (analogous to SV-, recombination- or selection-hotspots) within the genome of a lineage. This refined definition can enable comparative analyses across species, to take into account the methyltransferase and demethylase enzymes which have evolved over time to create adapted variants of methylome landscapes and the efficiency with which they can methylate target cytosines. ‘Methylation diversity,’ on the other hand, is a metric we propose for studying the concentration of genome-wide methylation. This represents the relative abundance of methylated sites in each species, normalized by genome-size, and is representative of overall methylation levels, perhaps indicating the varying reliance on this epigenetic mark for gene regulation across species (Figure 1).

This notion of epigenetic diversity allows us to address the question whether exposure to stress within a population can affect methylome or methylation diversity in a way so that it can be stably inherited to facilitate adaptive processes. Furthermore, we can ask whether or not the population genetic concept of genic allele frequencies (Gillespie, 2004) can also be extended to methylome or methylation landscapes. One would assume that accessions within a population would have less methylation diversity, but more methylome diversity. This outcome could be the result of a mechanism aiming to maintain the balance of the species-specific epigenome, but to moderately re-organize the target cytosines under a particular context, or the extent of methylation in each cell, to fine-tune gene regulation against the stress. For example, within different strains of the rice genome that have been independently domesticated over several generations, methylome diversity is correlated with bursts of transposition in the MITE family *mPong* (Lu et al., 2017). Methylation diversity can be more apparent between species - for example two angiosperms belonging to the *Brassicaceae* family - *Eutrema salsugineum* and *Conringia planisiliqua* completely lack gene-body methylation (Bewick and Schmitz, 2017; Bewick et al., 2017), or others such as rice which harbors an increased density of transposable-elements, necessitating heavy methylation for silencing (Zemach et al., 2010; Choi and Purugganan, 2018). This metric thus gives an insight into how epigenetic networks have evolved across species, tailor-made to their underlying genome structures.

In this article, we reviewed the role of active transposable elements in reshaping epigenetic landscapes through reintegration with potential long-term effects on subsequent generations. While various environmental stresses have been associated with particular transposon families, we believe that determining potentially mobile TE families and their copy number variation between ecotypes and species will become the new focus of large-scale epigenomics studies. For this purpose, a new generation of *de novo* TE annotation and detection tools are required that do not focus on classic annotation of all types of repeats, remnant TEs, co-opted sequences, and active elements (Goerner-Potvin and Bourque, 2018; Lanciano and Cristofari, 2020), but rather specialize on the detection of intact and potentially mobile TE families in *de novo* assembled genomes derived from long-read sequencing technologies (Drost, 2020). Being aware of the distinct TE families and their respective activation cues in each strain or species may be vital for determining competitive TE interactions during hybrid generation and cross-breeding.

## Discussion

Plants have widely diversified in their ability to colonize varying environments, characterized by a plethora of climatic variables such as temperature, precipitation, soil nutrition, pathogens, water availability and many more.

A large body of work has focused on understanding how these factors influence plant survival and adaptation across generations. In the wild, plants are exposed to multiple stresses in parallel, in addition to circadian and seasonal climatic changes. This cross-adaptation principle whereby exposure to one stress can prepare defense pathways against another related stress requires further attention. At the physiological level, several combinatorial stress-experiments have been carried out, examining epigenetic acclimatization efficiency against a particular stress (for example, Rivero et al., 2014) or to analyze concerted signaling mechanisms in response to multiple stresses (Zandalinas et al., 2020). In particular, future studies could characterize how cross-adaptation mechanisms utilize their epigenetic repertoire to shape an optimal gene regulatory network that can buffer or respond to various combinations of stresses in tandem.

**FIGURE 2:**
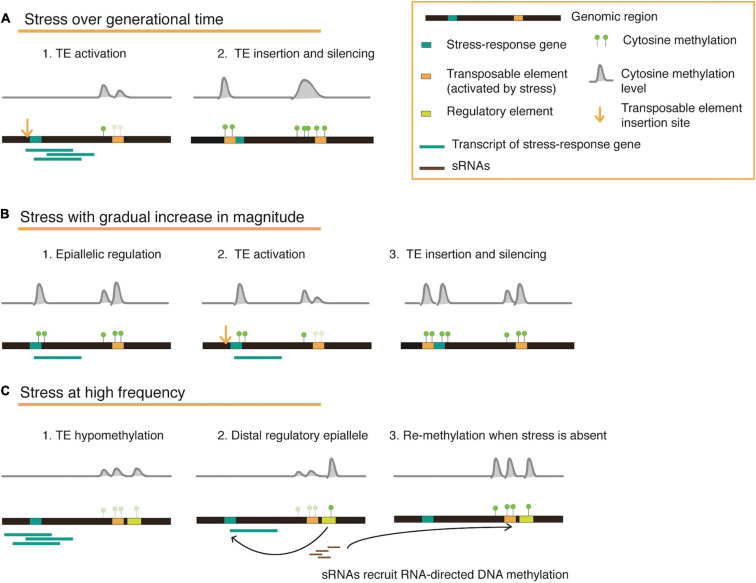
A conceptual model for transposable element and methylation dynamics illustrated in three different stress scenarios. The cartoons represent a genomic locus that houses a stress-response gene and a methylation-silenced transposable element (TE). When a stress is constantly applied over generations **(A)**, hypomethylation at the TE locus can result in activation and re-insertion proximal to a stress-response associated/stress-response repressor gene, eventually recruiting methylation marks to fix a new regulatory mechanism for long-term stability. When a stress increases in magnitude during the lifetime of a plant **(B)**, epialleles are first created to moderately regulate gene expression. To enable stronger gene expression changes, additional methylation changes are driven by activated TEs and their re-insertion. In situations where stress occurs repeatedly for short intervals **(C)**, plants might require epigenetic switches that can be easily tuned. This can be facilitated by the presence of a distal regulatory element flanking a TE, which can also be hypomethylated upon TE activation, thus altering expression of a gene detrimental for stress-response. When TE activation is brief and does not involve copy number increase, small RNAs are recruited for methylation (through the RdDM pathway) and can spread to the neighboring element, thus switching ‘off’ the regulation once again when the stress is absent.

On reviewing several published works that examine the plant epigenetic landscape under stress, we realize that responses and adaptation against stress occur in parallel on various physiological and epigenetic levels of a plant. The duration, magnitude, and frequency of the applied stress determines which level has a stronger influence on shaping gene function, enzyme activity, or epigenetic marks in a transgenerational context (Figure 2), and may further be influenced by the availability of nutrients (energy balance) structured in a complex response hierarchy. An optimal method to employ both genetic and epigenetic responses is the mobilization and controlled reintegration of transposable elements upon consistent stress exposure. Plants store a wealth of information within their intergenic regions in the form of TEs, which may be mobilized and inserted to specific regions that may house stress-response associated or stress-response repressor genes, or perhaps located in accessible chromatin and co-occur with certain histone variants. Eventually, these re-inserted TE copies may be silenced by DNA methylation to regulate transcription levels. Since each TE family may have unique roles depending on their stress-activators, simultaneous but concerted activation may also facilitate flexibility to recurring stresses, and eventually fixation (Figure 2).

A detailed understanding of the interplay between physiological and epigenetic mechanisms during the plant adaptation process will allow us to create more powerful plant engineering tools in response to rapid changes in global environmental conditions such as global warming. While several studies have examined the co-occurrence of physiological and epigenetic changes (Fang et al., 2017; Neves et al., 2017), decoding the temporal hierarchy of interacting pathways at a high resolution and testing the heritability of these induced changes can enable better understanding of epigenetic preparation to future stress responses in plants. Gene engineering tools such as CRISPR/Cas9 are promising solutions for enabling the engineering of knockouts of key genes/enzymes for improved adaptability in several plant species. Recently, these tools have also been engineered to precisely manipulate locus-specific methylation levels (Gallego-Bartolomé et al., 2018, 2019; Papikian et al., 2019). Yet, this approach poses the limitation of inducing evolutionary trade-offs with other traits or compensatory mechanisms such as the activation of alternative pathways or undesired epigenetic remodeling at distant loci. We suggest that harnessing particular natural variants of the epigenetic toolkit derived from already adapted ecotypes in combination with controlled transposon activation will provide a more general plant engineering methodology. This new engineering methodology is capable of shaping entire plant epigenomes that at the same time have to co-adapt to various new stress conditions resulting from rapid environmental changes such as global warming.

Physiological responses to stress are well characterized mostly providing survival buffers for rhythmic events within a particular range of environmental conditions. In contrast, the epigenomic responses of a plant can not only regulate seasonal or circadian events (such as histone-methylation mediated transcriptional silencing of the *FLOWERING LOCUS C* (*FLC*) gene in winter-annual *Arabidopsis* accessions (Berry and Dean, 2015), but can also simultaneously rewire entire gene networks and transmit this change to subsequent generations thereby enabling long-term adaptation to completely new environmental conditions.

This notion allows us to redefine plant stress adaptation as a process that combines epigenetic and genetic mechanisms to restructure gene regulatory networks and maintain genome integrity. Such network restructuring events create a fast response to a qualitatively changing environment. On a population level, variants of restructured networks within individual plants are then selected to reduce genome destabilization and to buffer negative outcomes for reproductive fitness. In contrast, the rewiring of gene networks induced by physiological stress responses will always remain within the dynamic range set by the genetic and epigenetic levels and therefore reflects a static buffering mechanism with little potential to induce transgenerational adaptive change. On an evolutionary scale, it is the epigenetic level that interfaces between the environmental and genetic levels, thereby creating a transgenerational buffer while the physiological level represents the buffer zone that determines the adaptive flexibility of a plant species within a generation.

Applying the framework developed above, we predict that seasonal/cyclical or only slight changes to environmental conditions will have small effects on the epigenetic landscape in healthy non-starved plants and are less likely to be transmitted transgenerationally, whereas accelerating geological trends over time can be strong enough to overcome the physiological barrier and will induce significant changes to the genetic and epigenetic landscape that in turn will be exposed to natural selection and adaptation via transgenerational inheritance.

Our hypothesis further predicts that during environmental stress induction, the physiological-, epigenetic-, and genetic- levels follow a hierarchical principle of organization whereby each respective zone, gradually buffers the environmental impact over the long term. This principle generates a feedback loop between the physiological level and the epigenetic level such that environmentally induced epigenetic changes will occur most dramatically when physiological responses are insufficient whereas genetic changes are only inherited by subsequent generations when the epigenetic silencing marks are sufficiently erased leaving the genome exposed to structural variation and TE bursts.

Together, we postulate that rapid changes in global environmental conditions such as climate change in the coming years will require a new mode of plant engineering based on the control of methylation landscapes and transposon activation that can reshape entire gene regulatory networks and pathways in tandem to induce novel traits and physiological robustness to the newly emerging environmental conditions. Stabilizing these co-adapted traits transgenerationally may become the new focus of epigenetics and plant biotechnology research. Studies focusing on predicting the long-term effects of rapid environmental changes based only on a few stress variables (e.g., temperature and/or precipitation) may therefore largely overestimate the robustness of temperature/precipitation adapted ecotypes during climate change while underestimating the co-adapted traits that could either buffer or facilitate extinction events on the gene regulatory network level (e.g., compensatory pathways). As a result, future population epigenomics studies and plant engineering efforts will have to rely on new methodologies able to quantify all environmental variables in parallel to assess how they penetrate the entire gene regulatory network encoding co-adapted traits rather than relying only on a strict reductionist view of uncoupled stress variables. Epigenetic signatures such as the genome-wide distribution of DNA methylation marks or methylome/methylation diversity patterns can act as markers for selecting natural variants within populations or between ecotypes that reflect a stabilized and robustly adapted state of several co-evolved traits for which more realistic predictions of survival capacity in various environmental change conditions can be developed. The topologies of gene regulatory networks underlying particular epigenetic signatures will be more powerful predictors of future plasticity and adaptability in rapidly shifting environmental conditions than models focusing on individual SVs and their substitution frequencies. Ultimately, we envision that individuals within a population or ecotype lineages with robust co-adapted traits and gene-regulatory networks can then be used as starting material for artificial TE mobilization efforts, further fine-tuning beneficial traits to be fit for the new environmental demands.

## Data Availability Statement

The original contributions presented in the study are included in the article/supplementary material, further inquiries can be directed to the corresponding author/s.

## Author Contributions

Both authors listed have made a substantial, direct and intellectual contribution to the work, and approved it for publication.

## Conflict of Interest

The authors declare that the research was conducted in the absence of any commercial or financial relationships that could be construed as a potential conflict of interest. The handling editor declared a past co-authorship with one of the authors H-GD.
